# Actinomyces graevenitzii Detected in a Case of Endobronchial Actinomycosis Caused by Foreign Body Aspiration: A Case Report

**DOI:** 10.7759/cureus.107305

**Published:** 2026-04-18

**Authors:** Nao Hakamada, Hidesato Odaka, Kengo Shimada, Nanami Hatakeyama, Takuo Tokairin

**Affiliations:** 1 Clinical Training Center, Japanese Red Cross Akita Hospital, Akita, JPN; 2 Department of Respiratory Medicine, Japanese Red Cross Akita Hospital, Akita, JPN; 3 Bacteriological Laboratory, Japanese Red Cross Akita Hospital, Akita, JPN; 4 Department of Pathology, Japanese Red Cross Akita Hospital, Akita, JPN

**Keywords:** actinomyces graevenitzii, endobronchial actinomycosis, fish bone, foreign body aspiration, maldi-tof/ms

## Abstract

Pulmonary actinomycosis is a chronic respiratory infection caused by *Actinomyces*. *Actinomyces graevenitzii* is a rare species of *Actinomyces* with rare cases of infections. Herein, we report a case of endobronchial actinomycosis due to foreign-body aspiration in which *A.graevenitzii *was identified. The patient was a 48-year-old woman with a prior history of childhood asthma. She presented with a three-month history of hemoptysis, green sputum, and cough. Plain computed tomography of the chest revealed an elongated foreign body at the left inferior lobar bronchus, with infiltrative opacities extending from the foreign body to the peripheral regions and bronchial mucus plugs. Further inquiry revealed that she had choked while eating fish five years prior, followed by a persistent dry cough. She attributed this to her childhood asthma history. The foreign body retrieved during bronchoscopy showed histological features consistent with those of a bone. Thin, filamentous gram-positive bacilli were detected in the endobronchial lavage fluid and identified as *A. graevenitzii* by matrix-assisted laser desorption ionization time-of-flight mass spectrometry (MALDI-TOF/MS). Symptoms resolved after bronchoscopy. These findings indicate that *A. graevenitzii* can cause endobronchial actinomycosis following foreign-body aspiration and support the value of MALDI-TOF/MS for identifying the causative organism in endobronchial actinomycosis. The widespread adoption of MALDI-TOF/MS will facilitate the identification of previously difficult-to-identify *Actinomyces*, further improving understanding of their clinical characteristics and potentially informing treatment strategies.

## Introduction

Pulmonary actinomycosis is a chronic, suppurative, pulmonary or endobronchial infection caused by *Actinomyces* [1], with a prevalence of 15% in all actinomycosis cases [2]. *Actinomyces* species are anaerobic or facultative microaerophilic [2] and gram-positive bacilli belonging to the commensal flora of the human oropharynx, gastrointestinal tract, and urogenital tract [3].

Actinomycosis is generally considered an endogenous infection. Although the bacteria initially colonize the surface of the mucosa, they can reach deep tissues through disruption of the mucosal barrier caused by trauma, surgery, or foreign bodies [2]. Pulmonary actinomycosis is broadly classified into parenchymal, bronchiectatic, and endobronchial types, and the endobronchial type may result from bronchial foreign bodies contaminated with *Actinomyces* [4]. *Actinomyces* *israelii*, *Actinomyces naeslundii*, *Actinomyces odontolyticus*,* Actinomyces viscosus*, *Actinomyces meyeri*, *Actinomyces gerencseriae*, and *Actinomyces cardiffensis* have been identified as human pathogens [5,6]. *A. israelii* is the typical causative agent of pulmonary actinomycosis [7]. *A. graevenitzii* is a rare species identified in 1997, with limited information on its clinical characteristics [5]. Furthermore, to our knowledge, there are no reports of endobronchial actinomycosis caused by *A. graevenitzii* associated with foreign body aspiration.

Matrix-assisted laser desorption/ionization time-of-flight mass spectrometry (MALDI-TOF/MS) generates characteristic mass spectral fingerprints that serve as unique signatures for each microorganism, providing an effective tool for accurate microbial identification at the genus and species levels [8]. 

Herein, we report a case of endobronchial actinomycosis due to foreign-body aspiration in which *A. graevenitzii* was identified. MALDI-TOF/MS was useful in diagnosing this case.

## Case presentation

A 48-year-old woman presented to her local physician with complaints of bloody sputum, green sputum, and coughing that had persisted for three months. Her past medical history included childhood asthma. She had no regular oral medications. She had a history of smoking 10 cigarettes per day for 10 years, between the ages of 20 and 30 years. She worked as a cook at a restaurant. Family history included scirrhous gastric cancer in her mother. She was referred to the Japanese Red Cross Akita Hospital in Akita, Japan, for further evaluation and treatment.

On initial presentation, she was fully conscious, with a blood pressure of 114/78 mmHg, pulse rate of 61 beats per minute, temperature of 36.7°C, respiratory rate of 18 breaths per minute, and oxygen saturation of 98% on room air. Rhonchi were heard in the left lung field. No dental caries were present. No other significant physical findings were noted. Blood tests showed no elevated inflammatory markers, and other values were within normal limits (Table 1). 

**Table 1:** Laboratory investigation at the first hospital visit WBC, white blood cell; RBC, red blood cell; HGB, hemoglobin; PLT, platelet; ESR, erythrocyte sedimentation rate; Alb, albumin; TP, total protein; BUN, blood urea nitrogen; Cre, creatinine; Na, sodium; K, potassium; Cl, chloride; AST, aspartate aminotransferase; ALT, alanine transaminase; LDH, lactate dehydrogenase; CRP, C-reactive protein; IgG, Immunoglobulin G; IgA, Immunoglobulin A; IgM, Immunoglobulin M; IgE, Immunoglobulin E; CEA, carcinoembryonic antigen; CYFRA, cytokeratin 19 fragment; Pro-GRP, pro gastrin releasing peptide; T-SPOT, tuberculosis screening test; MAC, Mycobacterium avium complex; MALDI-TOF/MS, matrix-assisted laser desorption ionization time-of-flight mass spectrometry

Laboratory Investigation	Patient Value	Reference Range
WBC	5,700 /µL	3,300–8,600 /µL
Neutrophils	75.1%	41.2–69.7%
Lymphocytes	19.2%	22.1–46.9%
Monocytes	4.4%	4.1–9.6%
Eosinophils	0.5%	0.0–3.5%
Basophils	0.8%	0.0–1.1%
RBC	447×10^4^/µL	380–467×10^4^/µL
HGB	13.9 g/dL	11.9–14.3 g/dL
PLT	33.4×10^4^/µL	15.8–34.8×10^4^/µL
ESR	5 mm/h	3–15 mm/h
TP	7.2 g/dL	6.6–8.1 g/dL
Alb	4.1 g/dL	4.1–5.1 g/dL
BUN	17.5 mg/dL	8.0–20.0 mg/dL
Cre	0.53 mg/dL	0.46–0.79 mg/dL
Na	138 mmol/L	138–145 mmol/L
K	4.4 mmol/L	3.6–4.8 mmol/L
Cl	105 mmol/L	101–108 mmol/L
AST	24 U/L	13–30 U/L
ALT	14 U/L	7–23 U/L
LDH	194 U/L	124–222 U/L
CRP	0.08 mg/dL	0.00–0.14 mg/dL
IgG	1051 mg/dL	861–1747 mg/dL
IgA	395 mg/dL	93–393 mg/dL
IgM	78 mg/dL	50–269 mg/dL
IgE	110 IU/mL	0–170 IU/mL
β-D-Glucan	3.4 pg/mL	0.0–11.0 pg/mL
CEA	1.7 ng/mL	≤5.0 ng/mL
CYFRA	1.1 ng/mL	0.0–3.5 ng/mL
Pro-GRP	48.9 pg/mL	0–81 pg/mL
T-SPOT	(-)	(-)
*MAC* antibody	(-)	(-)
*Aspergillus* antigen	0.2	<0.5
*Aspergillus fumigatus* IgG antibody	<1.4 AU/mL	<5.0 AU/mL
*Aspergillus fumigatus* IgE antibody	<0.10 AU/mL	<0.35 AU/mL
General bacterial examination of sputum	Only oral commensal bacteria	
Sputum acid-fast bacilli test (smear)	(-)	(-)
Sputum acid-fast bacilli test (culture)	(-)	(-)
Sputum cytology	Class II, no fungi or malignant cells detected	
Bronchial lavage fluid culture	Gram-positive bacilli	(-)
MALDI-TOF/MS of Gram-positive bacilli detected in bronchial lavage fluid culture	*Actinomyces graevenitzii* (score ≥2.0)	score ≥2.0: species-level identification

**Figure 1:**
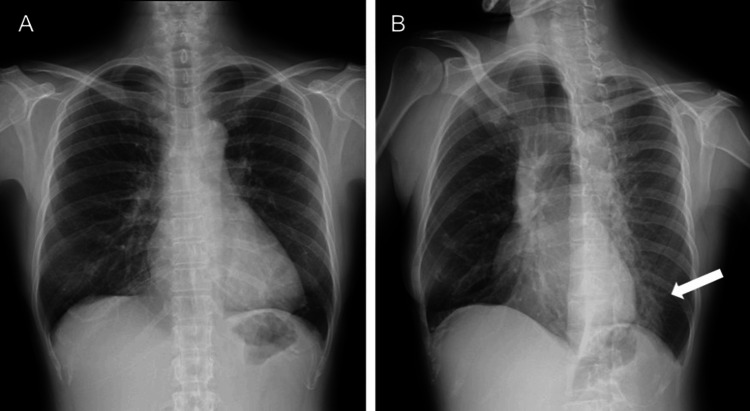
Chest plain radiographs at initial visit (A) Frontal view showing no significant decrease in lung field transparency. (B) Second oblique view showing a decrease in transparency in the left lower lung field (arrow).

General sputum bacterial culture, sputum acid-fast bacilli smear and culture, and sputum cytology showed no abnormalities. No obvious abnormalities were observed on the frontal view of the chest plain radiograph (Figure 1A). 

**Figure 2:**
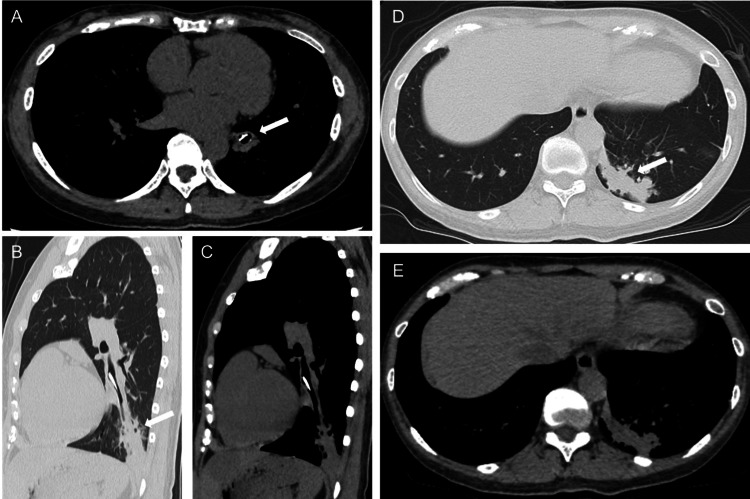
Initial chest computed tomography scan (A) A highly absorbent foreign body is identified at the left inferior lobar bronchus (arrow). (B) Infiltrative opacities extending from the foreign body to the peripheral region are observed (arrow). (C) No high-attenuation mucus is observed within the infiltrative opacities extending peripherally from the foreign body. (D) Club-shaped opacities suggestive of mucus plugs are observed (arrow). (E) No high-attenuation mucus is observed within the club-shaped opacities, suggestive of mucus plugs.

In the second oblique view, decreased transparency was noted in the left lower lung field (Figure 1B). Plain computed tomography of the chest showed a hyperdense foreign body at the left inferior lobar bronchus, with infiltrative opacities extending from the foreign body to the peripheral regions and bronchial mucus plugs (Figures 2A-2E). 

**Figure 3:**
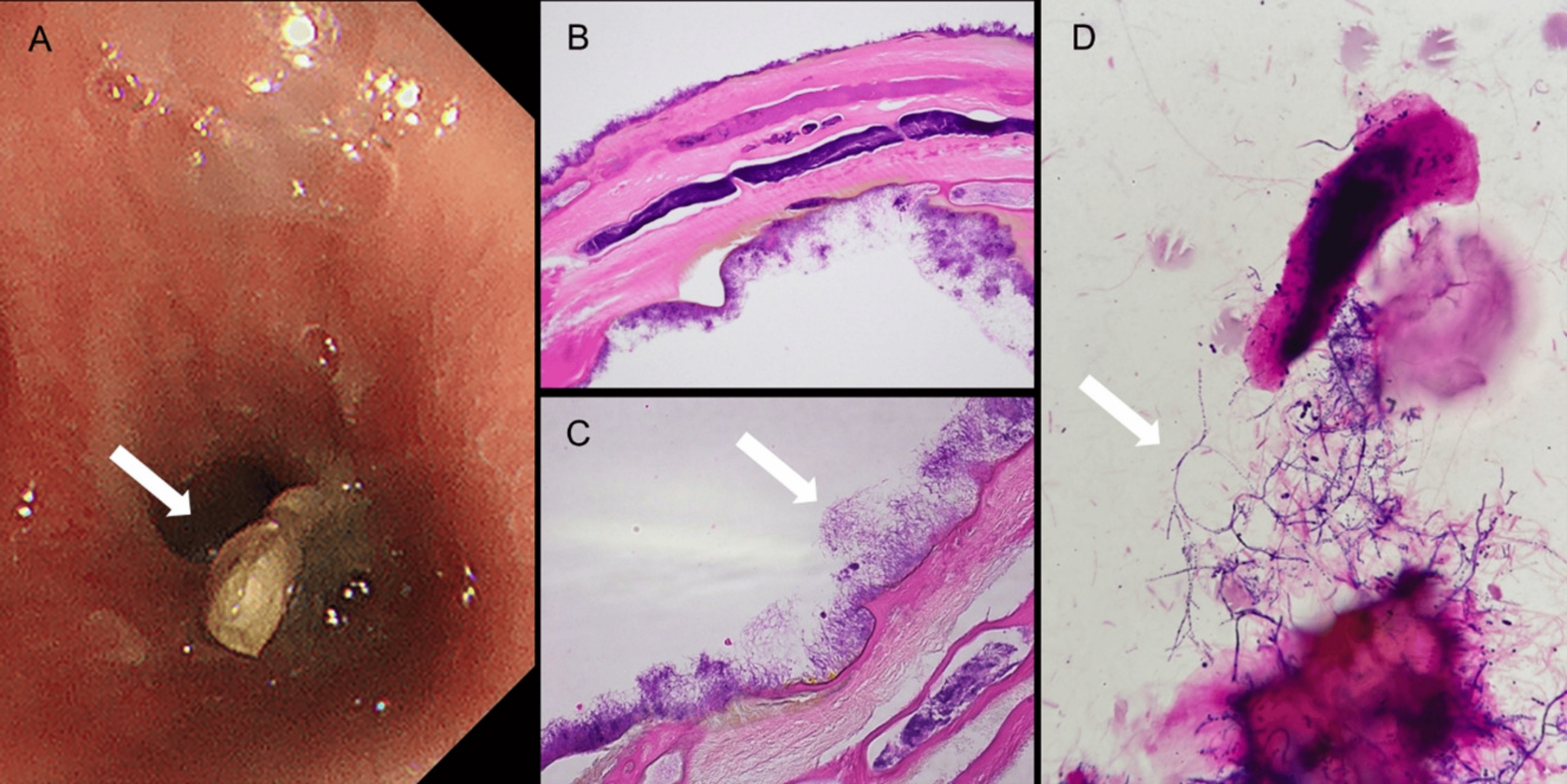
Bronchoscopy findings (A) A brownish foreign substance is observed at the left B10 entrance (arrow). (B) Hematoxylin and eosin-stained airway foreign body (×200). A structure consistent with a bone, composed of superficial cortical bone and deep medullary bone, is observed. (C) Hematoxylin and eosin-stained airway foreign body (×400). On the surface, thin filamentous gram-positive bacilli adhered in a radial pattern, intertwining with one another, presenting histological features consistent with those of *Actinomyces* (arrow). (D) Gram-stained bronchial lavage fluid (×1000). Thin, filamentous gram-positive bacilli are observed (arrow).

Differential diagnoses included obstructive pneumonia due to foreign body aspiration and allergic bronchopulmonary aspergillosis, based on the patient's history of childhood asthma and bronchial mucus plugs. Further inquiry revealed that five years prior, the patient had choked while eating fish, experienced severe coughing for several days, and subsequently developed a persistent mild dry cough. The patient attributed this to her history of childhood asthma. Bronchoscopy was performed under topical anesthesia with 2% lidocaine. A brownish-black foreign body was identified in the lumen of the left B10 bronchus, and it was removed using forceps (Figure 3A). 

The B10 bronchus was subsequently irrigated with saline. Histological examination of the extracted foreign body was consistent with bone (Figure 3B). On the surface, thin, thread-like gram-positive bacilli were attached in a radial pattern, intertwined with one another, and exhibited a histology consistent with that of *Actinomyces* (Figure 3C). Culture of the bronchial lavage fluid detected thin filamentous gram-positive bacilli (Figure 3D).

**Figure 4:**
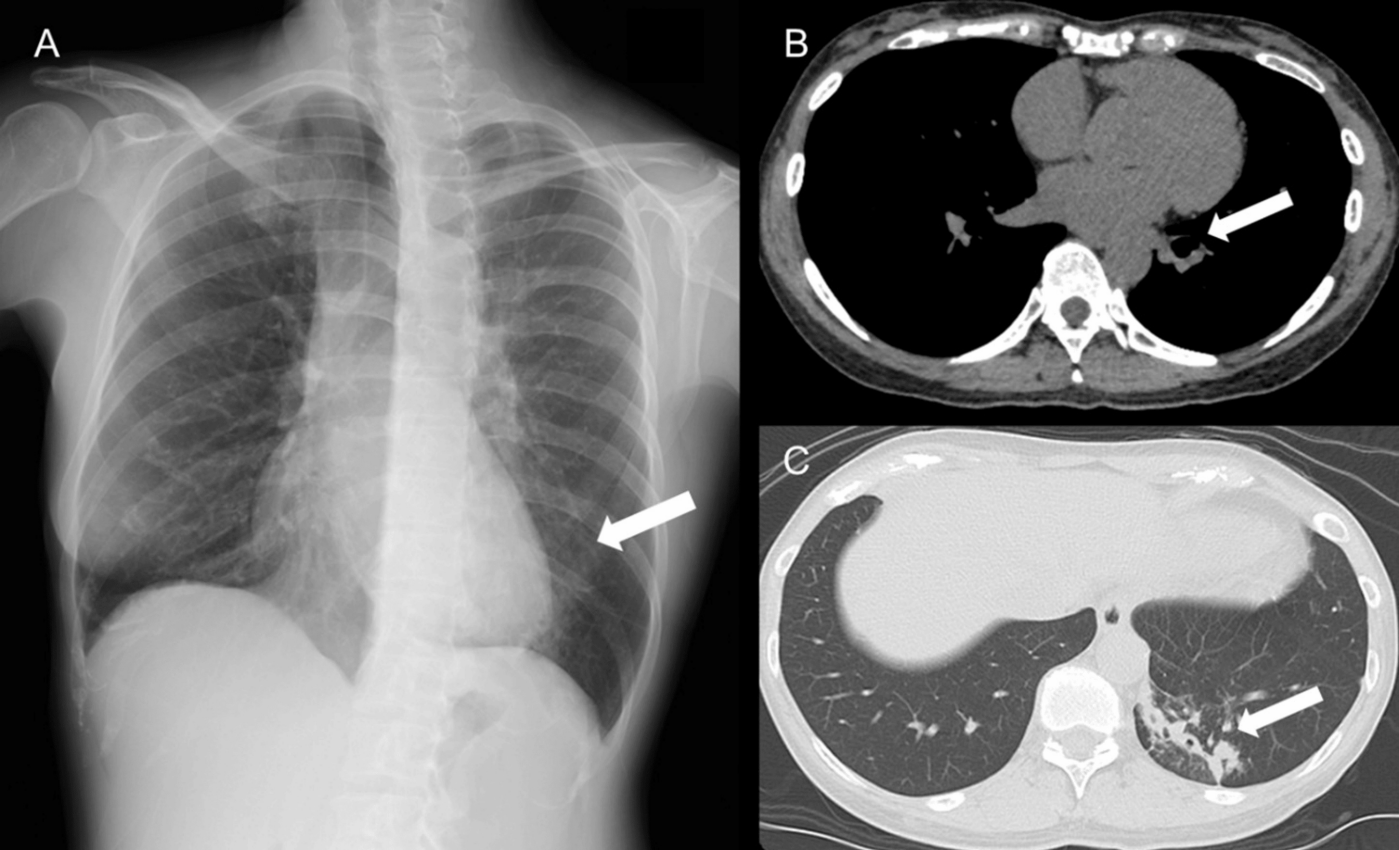
Imaging findings three weeks after bronchoscopy (A) Chest plain radiograph, second oblique view, three weeks after bronchoscopy. Improvement in the transparency of the left lower lung field is observed (arrow). (B, C) Chest plain computed tomography scan three weeks after bronchoscopy. No residual foreign body is detected after bronchoscopy, and the infiltrative opacities show a trend toward improvement (arrow).

The filamentous gram-positive bacilli were identified as *A. graevenitzii* by MALDI-TOF/MS at an external reference laboratory. Sulfur granules were not detected. Following bronchoscopy, symptoms resolved. A chest plain radiograph, second oblique view, taken three weeks after bronchoscopy, showed improved transparency in the left lower lung field (Figure 4A). 

Similarly, chest plain computed tomography three weeks after bronchoscopy revealed no residual foreign body and showed improvement in the infiltrative opacities (Figures 4B, 4C). Outpatient follow-up continues, and the patient has remained free of recurrence four months after bronchoscopy.

## Discussion

This case report highlights two significant findings: the detection of *A. graevenitzii* in a case of endobronchial actinomycosis caused by foreign body aspiration and the utility of MALDI-TOF/MS in diagnosis.

First, *e*ndobronchial actinomycosis may be caused by bronchial foreign bodies contaminated with *Actinomyces* [4]. When a foreign body contaminated with *Actinomyces* enters the airway, or when *Actinomyces* infects an existing airway foreign body, inflammation occurs in adjacent airways, leading to obstructive pneumonia in the peripheral regions [4]. Although several cases of respiratory infection caused by *A. graevenitzii* have been reported, to our knowledge, this is the first reported case associated with airway foreign bodies or accidental foreign body aspiration (Table 2) [1,3,5,7,9-14]. 

**Table 2:** Comparison of previously reported cases of Actinomyces graevenitzii infection with the present case COPD, chronic obstructive pulmonary disease; y, years; M, male; F, female

Report Year	Age, Gender	Diagnosis	Basic Background
2005 [9]	46Y, M	Disseminated co-infection with *Actinomyces graevenitzii* and *Mycobacterium tuberculosis*	Coronary artery disease, hypertension, chronic congestive heart failure, history of crack cocaine use
2007 [7]	52Y, M	Pulmonary actinomycosis	Crohn's disease, undergoing immunosuppressive treatment with Infliximab, budesonide, and 6‐mercaptopurine
2012 [1]	69Y, M	Organizing pneumonia with microabscesses	Smoking history of 60 pack-years
2012 [10]	38Y, F	Multiple lung abscesses	Inhalation of large amounts of dust
2014 [11]	35Y, M	Pulmonary abscess	Recent travel history to Sicily
2017 [12]	58Y, F	Pulmonary actinomycosis	Subclavian-jugular deep vein thrombosis, pulmonary thromboembolism, pulmonary hypertension, tricuspid regurgitation, bronchial asthma, missing teeth
2018 [5]	75Y, M	Pulmonary actinomycosis	History of pneumonia and Guillain-Barré syndrome, periodontal disease, 35-year smoking history (40 cigarettes/day)
2022 [3]	47Y, M	Pulmonary actinomycosis	Poor oral hygiene, 30-year smoking history (20 cigarettes/day), 20-year drinking history (150 mL/day)
2023 [13]	44Y, F	Pulmonary actinomycosis	Acquired bone marrow aplasia, systemic erythematosus lupus, undergoing immunosuppressive treatment with prednisolone and cyclosporin
2023 [13]	64Y, F	Pulmonary actinomycosis	Acute myeloid leukemia, undergoing consolidation chemotherapy with high-dose cytosine arabinoside
2025 [14]	75Y, M	Polymicrobial actinomycosis with *Actinomyces graevenitzii* and *Schaalia odontolytica*	COPD, bronchial asthma, and three years of inhaled corticosteroid use, history of pulmonary tuberculosis
2026 (Current case)	48Y, F	Endobronchial actinomycosis due to foreign body aspiration	Fish bone aspiration five years prior, pediatric asthma

Second, MALDI-TOF/MS proved helpful in diagnosing endobronchial actinomycosis caused by foreign bodies in the bronchial tract. Diagnosis can be challenging because pulmonary actinomycosis typically presents with nonspecific findings, including consolidation, mediastinal or hilar lymphadenopathy, atelectasis, cavitation, ground-glass opacities, and pleural effusion [6]. In this case, bronchial mucus plugs were also observed, making it necessary to differentiate this condition from other conditions, including inflammatory and infectious diseases (allergic bronchopulmonary aspergillosis and broncholithiasis), benign neoplastic processes (bronchial hamartoma, lipoma, and papillomatosis), and malignancies (bronchogenic carcinoma, carcinoid tumor, and metastases) [15]. Moreover, sulfur granules may be overlooked when the specimen volume is small [2]. When findings suggestive of actinomycosis are observed on culture or histopathological examination, *Actinomyces* species can be identified using MALDI-TOF/MS, which may aid in establishing the diagnosis.

For the Viridans group streptococci, species identification by MALDI-TOF/MS and classification into bacterial groups enabled characterization of clinical features specific to each group [16]. Similarly, within *Actinomyces*, the accumulation of cases involving species that were previously difficult to identify, made possible by MALDI-TOF/MS, has the potential to contribute to our understanding of the clinical characteristics and pathogenicity of these species as well as to the development of treatment strategies. Furthermore, because *Actinomyces* exhibits high resistance rates to certain antibiotics and resistance is species-dependent, identification to the species level is desirable [17]. To the best of our knowledge, although *A. graevenitzii* has been identified by MALDI-TOF/MS, it has not been identified in cases of foreign body aspiration (Table 3) [1,3,5,7,9-14]. 

**Table 3:** Bacterial identification methods in previously reported cases of Actinomyces graevenitzii infection. OCR, polymerase chain reaction; BALF, bronchoalveolar lavage fluid; MALDI-TOF/MS, matrix-assisted laser desorption ionization time-of-flight mass spectrometry; 16S rRNA, 16S ribosomal RNA.

Report Year	Age, Gender	Specimen	Microbial Species Identification Methods
2005 [9]	46Y, M	Sputum	PCR (16S rRNA sequencing)
2007 [7]	52Y, M	Bronchial lavage fluid	Not reported
2012 [1]	69Y, M	Thoracoscopic lung biopsy specimen	PCR (16S rRNA sequencing)
2012 [10]	38Y, F	BALF	PCR (16S rRNA sequencing)
2014 [11]	35Y, M	BALF	Not reported
2017 [12]	58Y, F	Lymph node biopsy specimen	Not reported
2018 [5]	75Y, M	BALF	MALDI-TOF/MS PCR (16S rRNA sequencing)
2022 [3]	47Y, M	BALF	MALDI-TOF/MS
2023 [13]	44Y, F	BALF	MALDI-TOF/MS
2023 [13]	64Y, F	BALF	MALDI-TOF/MS
2025 [14]	75Y, M	Bronchial lavage fluid	Mass spectrometry
2025 (Current case)	48Y, F	Bronchial lavage fluid	MALDI-TOF/MS

In this case, because the symptoms improved after bronchoscopy, we chose careful observation without treatment and did not administer antibiotics. There is a report of endobronchial actinomycosis resolving spontaneously after bronchoscopy [18]. This report suggested that bronchoscopic removal of bronchial mucus plugs and drainage of the bronchial lumen may create an aerobic environment at the lesion site, potentially contributing to symptom improvement [18]. We considered a similar course in this case. Careful follow-up is planned to monitor for recurrence.

## Conclusions

*A. graevenitzii* was detected in a case of endobronchial actinomycosis caused by foreign body aspiration. MALDI-TOF/MS has been proven useful for diagnosing endobronchial actinomycosis caused by foreign bodies in the bronchial tract. Further studies are needed to determine the frequency of “hidden” endobronchial actinomycosis due to *A. graevenitzii* and whether MALDI-TOF/MS can help identify such cases.
